# Insulin resistance, impaired glucose tolerance and alpha-thalassemia carrier state

**DOI:** 10.1186/s40200-015-0129-2

**Published:** 2015-02-05

**Authors:** Adele Bahar, Ramin Shekarriz, Ghasem Janbabai, Roya Shirzad, Mohsen Aarabi, Zahra Kashi

**Affiliations:** Diabetes Research Center, Mazandaran University of Medical Sciences, Sari, Iran; Cancer center, Mazandaran University of Medical Sciences, Sari, Iran; Department of internal medicine, Imam Khomeini hospital, Razi street, Sari, Iran

**Keywords:** Alpha thalassemia, Insulin resistance, Impaired glucose tolerance, Pre-diabetes

## Abstract

**Background:**

This study was designed to determine relationship between the glucose metabolism disorder (the insulin resistance and the impaired glucose tolerance) and α-thalassemia.

**Methods:**

In this historical cohort study, 80Alpha-thalassemia carriers and 80 healthy people were enrolled. The participants had no diabetes familial history and the waist circumference and blood pressure were in normal range (waist circumference of less than 102 cm in men, 88 cm in women and blood pressure <120/80 mmHg). The serum insulin level, fasting blood glucose (after 12 hours fasting) and two-hour plasma glucose during an oral glucose tolerance test (2-h OGTT) were measured. Insulin resistance was estimated according to homeostasis model assessment method (HOMA). Chi-square test, independent sample *t*-test and the relative risk were used for data analysis.

**Results:**

According to FBS and OGTT results, the percentage of diabetes mellitus and pre-diabetes were 1.3% and 33.8% in Alpha-thalassemia carriers, respectively. The control group showed 2.5% diabetic and 13.8% pre-diabetic cases as well. The relative risk for the glucose tolerance impairment (diabetes and pre-diabetes) was 2.78 (95% CI: 1.31-5.88, P = 0.07).Six and a half percent of the Alpha-thalassemia group and 2.5% in the control group had 2.25 ≤ HOMAIR ≤ 3.59 (an intermediate state of Insulin sensitivity) p = 0.443. In the study, there was no subject with insulin resistance (HOMAIR >3.59).

**Conclusions:**

The possibility of risk enhancement of the impaired glucose tolerance (pre-diabetes and diabetes mellitus) in patients with α-thalassemia is almost three times greater than the normal population without relationship with insulin resistance. Diabetic and pre-diabetic Alpha-thalassemia carrier state is younger than the general population suffering of these disorders.

## Introduction

The insulin resistance was reported in association with hemoglobinopathies [1]. An iron overload and a deposition in the liver and also the pancreatic β cells damage are considered as the causes of the impaired glucose tolerance in patients who need frequent blood transfusions [1,2]. In addition,the iron turnover due to the hemolysis of the microcytic erythrocytes can result in the oxidative stress and the insulin resistance [3]. A positive relationship between the oxidative stress, the iron overload and the insulin resistance was observed in beta- thalasseamia major patients [4]. The prevalence of the impaired glucose tolerance and the diabetes mellitus in this group were reported 4-24% and 0-26% respectively [5] in line with serum ferritin concentration [6]. The insulin resistance can be seen in minor thalassemia without impairment glucose tolerance [7]. A retrospective study on 1,861 patients with thalassemia major (1954–1998) showed a decrease in the prevalence of the glucose metabolism disorders and an increase in the mean age at the diagnosis of diabetes over the years. Early treatment with the iron chelators was proposed as the cause of this decline [8]. In our pervious study on 164 patients including 82 β-thalassemia traits and 82 normal cases,we showed that the prevalence of IGT (Impaired Glucose Tolerance) and diabetes were higher in patients with thalassemia minor than the normal subjects and the risk of diabetes and insulin resistance state were two times [9]. The prevalence of diabetes and glucose intolerance in patients with thalassemia major and minor was reported greater than the normal people in another study as well [10].

The number of studies on the α-thalassemia trait and the glucose impaired metabolism is scanty. In a study carried out in 1996, the incidence of Gestational Diabetes Mellitus (GDM) was doubled in women with α-thalassemia trait(s) than women with the iron deficiency anemia [11]. Lao and L.F (2001) stated that the alpha-thalassemia trait was an important factor in the diagnosis of the gestational diabetes (OR 11.74, 95% CI 6.37-21.63) [12]. Given the extent of the α-thalassemia disease in the north part of Iran and the possibility of the impaired glucose metabolism in these subjects and also the lack of adequate research in this area, the study was an attempt to investigate the relationship between the glucose metabolism disorder (the insulin resistance and the impaired glucose tolerance) and α-thalassemia.

## Materials and methods

After obtaining the ethical board committee of Mazandaran University of Medical Sciences approval, this historical cohort study examined, 80 α-thalassaemia trait cases (hypo chromic microcytic anemia, without an iron deficiency anemia and a normal hemoglobin electrophoresis) referred to the Mazandaran thalassaemia Centre and also related to the hematology clinic in Sari, the capital of Mazandaran province in the north part of Iran were enrolled. The control group consisted of 80 normal subjects referring to the same clinics for a routine evaluation and they were negative about α-thalassaemia. The sampling method was Judgmental or a purposive sampling. The study period was from April until March 2011–2012. The inclusion criteria were summarized as follows:

1. Age range between 20 and 60.
2. Without history of kidney, liver and heart diseases, (such as advanced liver, renal and heart failure).
3. Not taking medicine that affects the glucose tolerance (such as steroids, second- generation antipsychotic).
4. No history of diabetes mellitus.
5. No history of diabetes in parents and/or siblings.
6. Having a normal blood pressure (systolic blood pressure <120 and diastolic blood pressure <80 mmHg).
7. Waist circumference of less than 102 cm in men and 88 cm in women (according to the 2001 IDF, NCEP: ATPIII criteria) [13].

The weight of participants was measured with a digital scale (Microlife ws80 Switzerland), ±100 gr accuracy without shoes and excess clothing. The height was measured in straight position without shoes by a ruler (with a precision of 1 mm), while the back of head, thoracic spine, buttocks, and heels (qua were together) touched the wall.

The BMI (body mass index) was calculated by dividing the weight in kg by the square of height in meters (kg/m2). The waist circumference was measured with a flexible tape placed on a horizontal plane at the level of the iliac crest (with an accuracy of 1 mm).

The Venous blood samples for the laboratory studies (FBS, AST, ALT, hsCRP, serum insulin level, and after fasting for 12 hrs) were collected from both groups. A standard oral glucose tolerance test (OGTT) with 75 g glucose was done in two study groups. In both groups, participants with abnormal FBS and BS2hpG (FBS from 100 to 125 and BS2hpp ≥ 140-199) were re-tested for FBS and BS2hpG. Serum FBS was analyzed by enzymatic calorimetric method using the glucose kit of Parsazmoon Co, Iran. AST (Aspartate amino transferase) and ALT (Alanine aminotransferase) were measured by calorimetric method using Biosystem kit, Spain and Hitachi917, Germany. The high sensitive C-reactive protein (hs-CRP) was measured by imminotorbidometry assay by Pars Azmoon kit, England, using Hitachi917 machine Germany. ELISA test with Hitachi auto analyzer using Monobind kit, USA was used to assess serum insulin levels.

Insulin resistance was estimated after measuring the level of fasting blood sugar (glucose oxidase method using enzymatic colorimetric glucose kits, Pars, Iran) and serum insulin by the *HOMA*_*IR*_ formula (HOMA: Homeostasis model assessment method) were calculated.

$$ \mathrm{H}\mathrm{O}\mathrm{M}{\mathrm{A}}_{\mathrm{IR}}=\mathrm{Fasting}\kern0.5em \mathrm{plasma}\kern0.5em \mathrm{insulin}\left(\mathrm{m}\mathrm{I}\mathrm{U}/1\right)\times \mathrm{Fasting}\kern0.5em \mathrm{plasma}\kern0.5em \mathrm{glucose}\left(\mathrm{m}\mathrm{m}\mathrm{o}/1\right)/22.5 $$

The participants were divided into three following categories:

1. HOMAIR < 2.24 as a sensitive one to insulin (insulin sensitive)
2. 2.24 ≤ HOMAIR ≤ 3.59 as an intermediate state (Intermediate)
3. HOMAIR > 3.59 as an insulin resistant [1]

The data was analyzed by using SPSS software. The mean standard deviation (SD), mode and percentiles were used for the purpose of descriptive analysis. The demographic and clinical characteristics were described as a proportion and a mean ± SD. The data analysis was performed by the Independent Student Sample *t*-test to compare the quantitative variables and the Chi-square test to compare the qualitative ones between the two groups. In addition, the relative risk was calculated. P-value of less than 0.05 was considered statistical significant level . The normal distribution of variables was confirmed by One-Sample Kolmogorov-Smirnov Test.

## Results

160 subjects in this study were studied. Out of 80 Alpha-thalassemia carriers state, 30 subjects (37.5%) were male and the rest were female and in the control group, both males and females consisted 40 cases (50%). (p = 0.11).

These two groups were matched for age, waist circumference, and blood pressure. The mean of the waist circumference in Alpha-thalassemia group was 87.1 cm (95% CI: 85.5 to 88.6) while in the control group, it was 88 cm (95% CI: 86.3 to 89.7), (p = 0.41), (the demographic characteristics are shown in Table 1). The mean age of subjects in the study was 32.5 ± 9.9 years, ranged between 20 to 58 and 20 to 55 in years old the Alpha-thalassemia and control groups respectively. The variables showed significant differences for AST, Hb, MCV and ferritin between two groups (see Table 2).

**Table 1:** **The Demographic characteristics in the exposure group (**α**-thalassemia) and people without exposure**

	**Case (n = 80)**		**Control (n = 80)**		P-value*
	**Mean**	**95% CI**	**Mean**	**95% CI**	
Age (Years)	33.2	30.9-35.5	31.8	29.7-33.9	0.354
Waist Circumference (cm)	87.1	85.5-88.6	88	86.3-89.7	0.414
Systolic Blood Pressure (MmHg)	112.6	111.1-114.1	111.2	109.7-112.7	0.182
Diastolic Blood Pressure (MmHg)	74.3	73.1-75.4	75.3	74.4-76.2	0.154

* Independent Student *t*-test.

**Table 2:** **The Distribution of some laboratory tests in the** α**-thalassemia and controls**

	**Case (n = 80)**		**Control (n = 80)**		P-value*
	**Mean**	**95% CI**	**Mean**	**95% CI**	
**AST (mg/dL)**	22	20.5 - 23.4	24.8	23.6 -26.0	**0.004**
**ALT (mg/dL)**	19.8	18.0 - 21.6	19.6	17.9 - 21.2	0.831
**Haemoglobin**	11.9	11.5 - 12.3	13.8	13.6 -14.1	<0.0001**
**MCV**	73.1	71.8 - 74.4	87.1	86.5 - 87.8	**<0.0001**
**Ferritin**	129.1	90.3 - 167.8	85.3	77.4 - 93.3	0.029**
**hsCRP**	2.66	2.14 - 3.17	2.43	2.12 - 2.74	0.46

*Independent Student *t*-test.

**the difference was significant after considering the age and gender as covariates in the comparison two groups by ANCOVA test.

The mean of fasting blood sugar (FBS) in the main group (93.7 ± 12.3 mg/dl) was higher than that in the control group (86.9 ± 12.4) (p = 0.001). The average blood sugar after OGTT (75 gr oral glucose) was 97.7 ± 25.6 mg/dl, whereas it was approximately 91.8 ± 23.6 mg/dl in the control group (p = 0.133). The mean of fasting insulin levels was 4.80 *micIU*/*ml* (95% CI: 4.28 - 5.33) and 4.77 *micIU*/*ml* (95% CI: 4.30 - 5.24) in the main and control group respectively (p = 0.924). The mean of HOMA_IR_was1.13 ± 0.62 in cases and 1.013 ± 0.50 in the controls (p = 0.283) .

**Figure 1:**
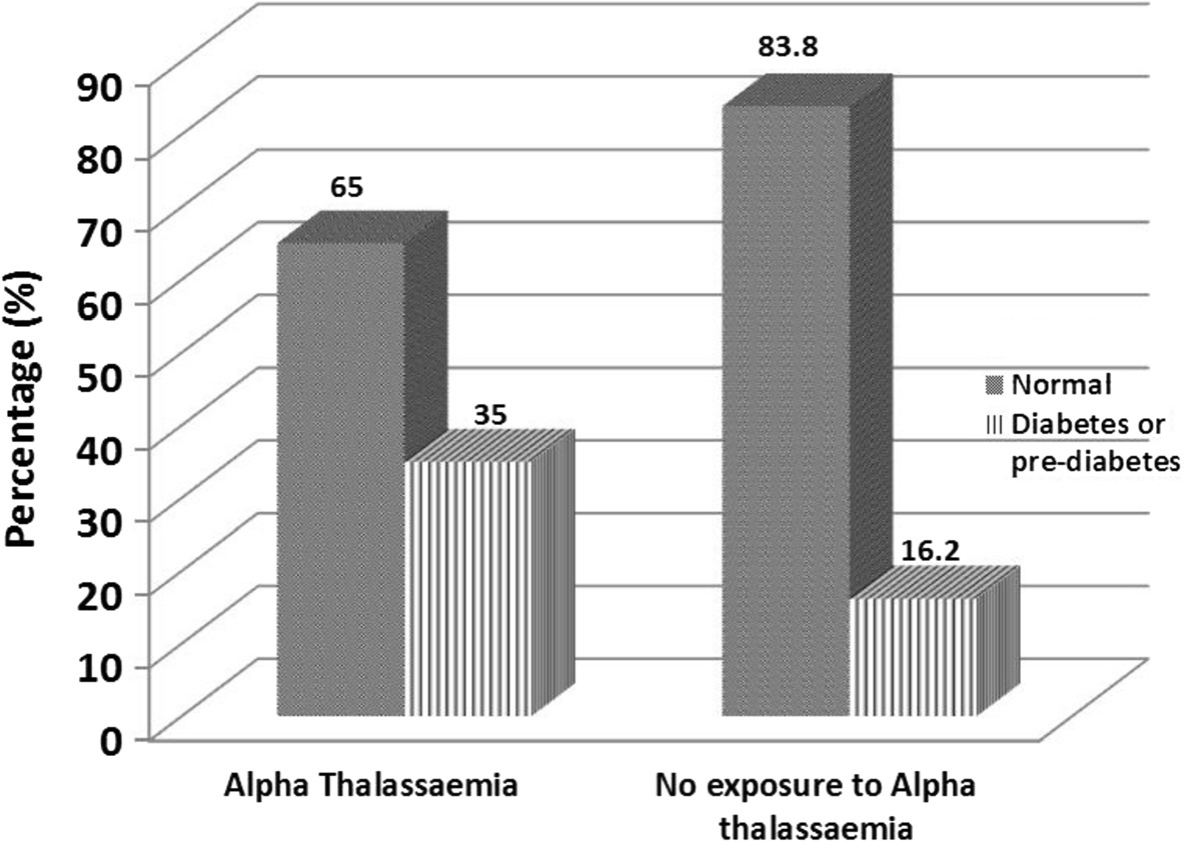
**The proportion of diabetes or pre-diabetes in the exposure group (α-thalassemia) and people without exposure (Pvalue = 0.007,Chi-square test).**

27 cases (33.8%) and 11 (13.8%) of the controls had IFG or IGT (pre-diabetic state). The risk of the pre-diabetes state was 3.16(95% CI: 1.44-6.69, P = 0.003) in α-thalassemia patients. One person (1.3%) in the case group and 2 people (2.5%) in the control group were diagnosed as a diabetic case (see Figure 1). The relative risk of the glucose tolerance impairment state (pre-diabetes or diabetes mellitus) was 2.78 (95% CI: 1.31 - 5.88), (p = 0.007) in the α-thalassemia compared to the control and the α-thalassemia carrier state patients were almost 3 times more at risk of impaired glucose tolerance (pre-diabetes and diabetes mellitus) than the normal state.

AST levels in the case sand control were 22 IU/L(95% CI: 20.5 - 23.4) and 24.8 *IU*/*L* (95% CI: 23.6 -26) respectively, (p = 0.004). Although the level of ALT and CRP were not significantly different between two groups, there was a positive correlation between hsCRP and ALT as well as ferritin (r = 0.3, p = 0.03 and r = 0.4, p = 0.001 respectively) only in alpha thalassemia group (see Table 2). According to HOMA-IR method, 6.5 percentage of main group and 2.5% of control was considered as intermediate state, (p = 0.443). No subject with insulin resistance was observed .there were no subject with insulin resistance (HOMAIR >3.59). 18 out of 27 patients (66.7%) with pre-diabetes state in Alpha-thalassemia carriers and 7 out of 11 pre-diabetic persons in the control group (63.6%) were less than 40 years old (p = 0.011).

## Discussion

The results show that the risk of the impaired glucose tolerance state (pre-diabetes or diabetes) is higher in the α-thalassaemia than the normal population, 2.78 (95% CI: 1.31 - 5.88), (p = 0.007) and the patients with α-thalassemia are almost three times more at risk. In a study conducted in Hong Kong by Lao and HO in 1996 on women with anaemia during pregnancy and its outcome, they found out that the incidence of the gestational diabetes in women with the α-thalassaemia trait doubled compared to the iron deficient pregnant women [11].

Lao and HO (2001) did another study on the pregnant women. 163 α-thalassaemia trait pregnant cases were examined. According to the results of oral glucose tolerance test (OGTT), the glucose tolerance was impaired in 62% of the α-thalassaemia Trait group and 14.7% of control group [12]. In present study, the prevalence rate of the impaired glucose tolerance (pre-diabetes and diabetes mellitus) in non-pregnant women with the age groups of 20 to 60 years was 35% and 16.3% in the α-thalassaemia and the control group respectively. The probability of diabetes and pre-diabetes state was 2.78 times in the α-thalassaemia group compared to the control. The prevalence of diabetes in alfathalassaemia was 1.3 percent which was considerably lower than the previous our pervious study on beta thalassaemia, 8.5% [9]. In contrast, the prevalence of pre-diabetic in α-thalassemia in the present study was 33.8% which was higher than the pre-diabetic prevalence in β-thalassaemia minor patients, 12.2% [9]. Perhaps a higher prevalence of diabetes than pre-diabetes in β-thalassemia is due to more hemolysis resulting in more inflammation of the liver in β-thalassaemia than α-thalassaemia. In both studies on alpha and beta thalassemia carrier state, pre-diabetes condition was higher than the normal population. In this study, the difference between the two groups (alpha thlassaemia and the normal individuals) based on fasting insulin levels and HOMA_IR_, was not statistically significant but in beta thalassaemia minor study, two patients (2.4%) in the case group and no patient in control (0%) had an insulin resistance. In a study on 17 normal glucose tolerance beta thalassaemia minor trait patients by Tong and colleagues (2002), fasting insulin levels were higher than control (p = 0.003) and the insulin resistance state was higher in the thalassemia trait group too (p = 0.004) [7]. The difference between these studies is some extent due to the size of the waist circumferences and exclusion of the cases with high waist circumferences and also cases at risk of metabolic syndrome in this study. The multiple research showed the correlation of liver inflammation and serum level of ALT,AST, ALP and Gamma glutamyltransferase (GGT) and also insulin resistance [4,6,14]. In Simvax study the oxidant stress and modulation of adipokines and intracellular signal transduction pathways were suggested as underlying molecular mechanisms of iron and diabetes risk [15]. In present study,there was a positive correlation between hsCRP and ALT as well as ferritin only in alpha thalassemia group but we didn’t find any relationship between insulin resistance and ALT, CRP and ferritin. Maybe there are other pathways which affect the glucose homeostasis in individuals with alpha thalassemia however, they are not still established and further research is required. Diabetic and pre-diabetic Alpha-thalassemia carriers are younger than the general population suffering from these disorders. Though the failure to identify significant difference between the HOMR-IR between the two groups in our study may be related to sample size, it seems that the higher risk of the impaired glucose condition in alpha-thalassemia carriers state is not directly due to the insulin resistance and the mechanism of the disorder should be taken into consideration.

The limitation of the study was duo to the lack of evaluation of the GGT serum level and oxidative stress and other specific inflammatory markers.

## Conclusion

Given the high prevalence of α-thalassaemia in the north part of Iran and risk enhancement of the impaired glucose tolerance in this group, it is recommended that the α-thalassaemia state should be considered as one of the diabetes risk factors. It is also advised that this group is screened for IGT and diabetes at younger age compared to the normal population in order to detect the disease as early as possible and also to take the preventive interventions.

Finally, we recommend further research with larger sample size is done to compare the inflammatory factors, the oxidative stress markers and the haemolysis status between α-thalassemia and β-thalassaemia.

### Consent

Written informed consent was obtained from the patient for the publication of this report.
